# Supplementary material for: The global impact of COVID-19 on tuberculosis: A thematic scoping review, 2020–2023
Source: PLOS Glob Public Health. 2024 Jul 3;4(7):e0003043. doi: 10.1371/journal.pgph.0003043 (PMC11221697; doi:10.1371/journal.pgph.0003043)
Supplement: S1 Text — (DOCX) [file pgph.0003043.s001.docx]

S1 Text: Scoping Review Protocol

**Published at:** [**https://doi.org/10.6084/m9.figshare.24566842**](https://doi.org/10.6084/m9.figshare.24566842)

**(JBI Evidence Synthesis)**

Protocol for “Global Impact of COVID-19 on Tuberculosis: A Scoping Review and Thematic Analysis, 2020-2023”

Michael H. Marco, PhD, MPH^1^; Sevim Ahmedov, MD, MPA^1^, Kenneth G. Castro^1,2^

1. TB Division, Office of Infectious Diseases, Bureau for Global Health, United States Agency for International Development, Washington, District of Columbia, United States of America
2. Rollins School of Public Health and School of Medicine, Emory TB Center, Emory University, Atlanta, Georgia, United States of America

**Abstract**

**Objective:** The objective of this scoping review is to understand the extent of evidence and thematic issues surrounding COVID-19’s global impact and effect on tuberculosis (TB), from the clinical cascade to patient-centered care and psychosocial issues, using publications and grey literature.

**Introduction:** Numerous publications exist on the deleterious impact of COVID-19 on TB, yet almost all focus on one issue. Hence, the rationale for this review is to offer a 360-degree global snapshot that will categorize, digest, and elucidate the issues into prominent themes for developing policy recommendations to inform and stimulate preparedness, response, recovery, and resilience for multiple facets of TB globally.

**Inclusion criteria:** Participants will be people infected with or at risk of TB, their families, and health care providers. The primary inclusion criteria stipulate that publications have COVID-19 as the sole exposure and TB as the sole outcome to observe a direct causal pathway. We included publications with original research––qualitative and quantitative–and letters to the editor, editorials, and reviews with the requirement that letters and editorials include original data or substantive recommendations.

**Methods:** Publications written in English between 1 January 2000 and 30 April 2023 were captured, starting 1 May 2023, from three search engines: PubMed, EBSCO, and Google Scholar, using PubMed MeSH terms related to SARS-CoV-2/COVID-19, TB, and health services. Publications retrieved from the search were uploaded to COVIDENCE software, where two authors, with a third as an arbitrator, screened titles and abstracts. Websites of global health multilateral organizations were combed to identify pertinent grey literature. Citation searching from publications identified references not appearing in search engines. Our methodological approach employs PRISMA extension for scoping reviews (PRISMA-ScR) guidance and Joanna Briggs Institute methodology. Data were extracted by all three authors using a thematic data extraction tool developed by the review team. Descriptive variables extracted included author(s), geography, publication year, publication type, and perceived theme(s). Publications with their respective themes are presented in tabular form and accompanied by a narrative summary.

**Results:** Of 1,755 screened publications, 176 (10%) covering 39 countries over 41 months met the inclusion criteria. We established ten principal themes encompassing TB’s care cascade, patient-centered care, psychosocial issues, and health services: 1) case-finding and notification (n=45; 26%); 2) diagnosis and laboratory systems (n=19; 10.7%) 3) prevention, treatment, and care (n=22; 12.2%); 4) telemedicine/telehealth (n=12; 6.8%); 5) social determinants of health (n=14; 8%); 6) airborne infection prevention and control (n=8; 4.6%); 7) health system strengthening (n=22; 13%); 8) mental health (n=13; 7.4%); 9) stigma (n=11; 6.3%); and 10) health education (n=10; 5.7%). Thematic-based policy recommendations for TB recovery and resilience are included.

**Conclusions:** The 41-month timespan allowed for amassing publications with data from multiple SARS-CoV-2 pandemic waves: Wuhan-Hu-1, Delta, and Omicron variants. This thematic scoping review establishes that, regardless of a country’s geography or income level, there were resonating generalizable themes on the deleterious impact of the COVID-19 pandemic on multiple areas of TB. A significant limitation was the heterogeneity of publications within themes.

**Introduction**

The global spread of SARS-CoV-2, the cause of COVID-19, likely derailed the promise of and trajectory for ending TB in this decade. COVID-19 wiped out over 12 years of hard-fought gains, such as increased TB detections and decreased TB-related mortality [1]. It is estimated that there will be 4,702,800 additional TB cases and 1,044,800 TB deaths worldwide between 2020 and 2025 due to the disruptions in TB detection and treatment during lockdowns and the prioritization of COVID-19 services [2].

We conducted a thematic scoping review to explore the impact, effect, and aftermath of COVID-19 on all aspects of TB services. Moreover, this review sought to understand and interpret the scope of resonating themes surrounding the pandemic’s impact on TB. Based on our analysis, we expanded the traditional model of a scoping review by offering policy recommendations that align with the principal themes.

Over the past three years, there has been a significant amount of literature from around the globe on specific TB issues (e.g., case-finding and notification) in which COVID-19 has negatively impacted TB services and psychosocial problems. Review articles have done well to gather and discuss several topics in the field [3-5]. Yet, many do not offer a holistic view of the clinical cascade to patient-centered care provision. Thus, there is a need for a comprehensive scoping review using a 360-degree lens to identify global publications detailing COVID-19’s multiple negative impacts across all aspects of TB over three and one-half years. To capture a wide range of publications for a thematic analysis, we included editorials, letters to the editor, conference proceedings, and reviews to accompany original research publications.

A scoping review was most suitable for this topic because it aids in identifying essential characteristics or factors (e.g., themes) related to a concept and employs a knowledge synthesis approach (e.g., categorization) to a research area [6]. A preliminary search of PubMed, EBSCO, and Google Scholar was conducted, and no multi-topic, comprehensive scoping, or systematic reviews on this topic were identified. For single issues/themes, we identified two rapid reviews––one on COVID-19’s impact on TB prevention [7] plus another on COVID-19’s impact on TB infection control [8]––and a scoping review on the pandemic’s impact on TB health systems [9].

The objectives of this thematic scoping review are to understand and appreciate the global impact of COVID-19 on TB, interpret the scope of resonating themes, and offer policy recommendations to stimulate TB recovery and future pandemic preparedness. This was accomplished by analyzing original research, letters to the editor, editorials, and reviews that discuss COVID-19 (as the sole exposure) as the impact, effect, or consequence of any aspect of TB (as the sole outcome).

Letters to the editor and editorials were required to contain original data or substantial recommendations. We will exclude 1) published abstracts; 2) magazines; 3) online pre-publications that were never published; and 4) literature focusing on the clinical sequelae of COVID-19 and TB co-infection, or dynamics of co-infection with TB and COVID-19 and other illnesses (e.g., HIV, diabetes) that could be confounders or mediators.

**Review question**

What are the principal themes resonating from a comprehensive literature analysis on COVID-19’s impact, effect, and aftermath on multiple facets of TB that can inform policy recommendations to assist in TB resilience, recovery, and future pandemic preparedness?

**Keywords**

COVID-19, healthcare, health systems, pandemic, SARS‐CoV‐2, tuberculosis

**Eligibility criteria**

***Participants***

Participants in the scoping review were people with TB, survivors of TB, individuals at high risk of acquiring TB (e.g., the malnourished), caregivers, families, healthcare providers, and members of civil society*.*

***Concept***

COVID-19 was the sole exposure on the causal pathway to any aspect of TB as the sole outcome. We excluded literature focusing on the clinical sequelae of COVID-19 and TB co-infection or the dynamics of co-infection with TB and COVID-19 and other illnesses (e.g., HIV, diabetes) that could be a confounder(s) or mediator(s) in the causal pathway.

***Context***

We sought to capture publications from low-, middle-, and high-income countries with literature meeting the inclusion criteria. This approach enabled us to determine the global generalizability of the principal themes from diverse cultures and patient populations.

***Types of Sources***

This scoping review considered experimental and quasi-experimental study designs, such as randomized and non-randomized controlled trials and interrupted time-series studies. Analytical observational studies, including prospective and retrospective cohort studies, were considered for inclusion. This review also drew from descriptive observational study designs, including case series, individual case reports, and descriptive cross-sectional studies for inclusion. Modelling studies were included.

In addition, letters to the editor, editorials, conference proceedings, reviews, and grey literature that meet the inclusion criteria were considered.

***Methods***

This thematic scoping review was conducted in accordance with PRISMA Extension for Scoping Reviews (PRISMA-ScR) guidelines [10] and Joanna Briggs Institute methodology [11].

***Search strategy***

The search strategy aimed to locate applicable published and grey literature. Our review team met twice in April 2023 to discuss the review's scope and develop search terms. The leading search terms included COVID-19/SARS-CoV-2, tuberculosis, and healthcare/health systems (S2 Text).

Only publications written in English were included. Studies published from January 1, 2020, to April 30, 2023, were included. This timeframe coincides with the advent of COVID-19 and the day before the search commenced.

The databases searched include PubMed, EBSCO, and Google Scholar. Sources of grey literature searched included global health multilateral websites from the World Health Organization, The Global Fund to Fight HIV, TB, and Malaria, and The International Union Against Tuberculosis and Lung Disease.

We searched citations of the captured publications to identify eligible literature missed by the databases.

Identified citations were uploaded to EndNote 20 (Clarivate Analytics, PA, USA) and transferred to COVIDENCE systematic review software to screen and remove duplicates. MHM and KGC independently conducted a screening of titles and abstracts. The full text of selected citations was independently assessed in detail by MHM and KGC against the inclusion criteria. Any disagreements between the MHM and KGC at each stage of the selection process were arbitrated and resolved by the co-author, SA.

The results of the search are detailed in a schema (Primary text: Fig 1), and the study inclusion process and thematic capture for analyses are described in the “Methods” and “Results” sections of the primary text.

***Data Extraction***

Our draft extraction tool will be provided (S1 Table). It will be modified and revised as necessary while extracting data from each publication. The deviation from the PRISMA ScR will be required because of the heterogeneous array of publications (i.e., original research, editorials, and letters to the editor), many of which will be void of data. Hence, our tool is best suited for extracting a descriptive thematic analysis. The data extracted will include descriptive details about the author(s), year, geographic location, type of publication, principal theme, and two ancillary themes because of substantial, pertinent discussions on additional underlying causes or solutions.

***Data Analysis and Presentation***

The extracted and analyzed publications will be alphabetically listed in an unstipulated number of thematic tables (e.g., prevention, treatment, and care) (S3 Table). The thematic tables will be designed to include descriptive variables, including author(s), year, publication type, and country, region, or continent. In the primary texts, the “Results” will contain a narrative summary by theme with 1) a broad overview; 2) a publication(s) with global data; 3) a publication presenting a single country’s experience; 4) a publication(s) that delves into underlying causes or novel solutions*.*

**Acknowledgments**

We thank Cheri Vincent, Tara Ornstein, Amy Bloom, and YaDiul Mukadi for discussions regarding the scope of the review, assistance in developing the search string, and creating the draft extraction tool; Tracy Swan for editorial assistance; Margie Davis and David Pieribone for graphic assistance with Figs 1 and 2; Marcus Renick for graphic assistance with supplement material; and COVIDENCE for offering complimentary software usage.

**Funding**

Source of funding for the review: United States Agency for International Development (USAID).

**Conflicts of Interest**

There are no conflicts of interest in this project.

**References**

1. Sahu S, Ditiu L, Sachdeva KS, Zumla A**.** Recovering from the Impact of the Covid-19 Pandemic and Accelerating to Achieving the United Nations General Assembly Tuberculosis Targets. Int J Infect Dis. 2021;113 Suppl 1:S100-3. doi: 10.1016/j.ijid.2021.02.078.

2. Cilloni L, Fu H, Vesga JF, Dowdy D, Pretorius C, Ahmedov S, et al. The potential impact of the COVID-19 pandemic on the tuberculosis epidemic a modelling analysis. EClinicalMedicine. 2020;28:100603. doi: 10.1016/j.eclinm.2020.100603.

3. Zimmer AJ, Klinton JS, Oga-Omenka C**.** Tuberculosis in times of COVID-19. J Epidemiol Community Health. 2022;76(3):310-6. doi: 10.1136/jech-2021-217529.

4. McQuaid CF, Vassall A, Cohen T, Fiekert K, White RG**.** The impact of COVID-19 on TB: a review of the data. Int J Tuberc Lung Dis. 2021;25(6):436-46. doi: 10.5588/ijtld.21.0148.

5. Klinton JS, Heitkamp P, Rashid A, Faleye BO, Win Htat H, Hussain H, et al. One year of COVID-19 and its impact on private provider engagement for TB: A rapid assessment of intermediary NGOs in seven high TB burden countries. J Clin Tuberc Other Mycobact Dis. 2021;25:100277. doi: 10.1016/j.jctube.2021.100277.

6. Arksey H, O'Malley L**.** Scoping studies: towards a methodological framework. Int. J. Soc. Res. Methodol. 2005;8(1):19-32. doi: 10.1080/1364557032000119616.

7. Jeong Y, Min J. Impact of COVID-19 pandemic on tuberculosis preventive services and their post-pandemic recovery strategies: a rapid review of literature. J Korean Med Sci. 2023;38(5):e43. doi: 10.3346/jkms.2023.38.e43.

8. Chapman HJ, Veras-Estévez BA**.** Lessons learned during the COVID-19 pandemic to strengthen tb infection control: a rapid review. Glob Health Sci Pract. 2021;9(4):964-77. doi: 10.9745/ghsp-d-21-00368.

9. Dlangalala T, Musekiwa A, Brits A, Maluleke K, Jaya ZN, Kgarosi K, et al. . Evidence of TB services at primary healthcare level during COVID-19: a scoping review. Diagnostics (Basel). 2021;11(12):2221. doi: 10.3390/diagnostics11122221.

10. Tricco AC, Lillie E, Zarin W, O'Brien KK, Colquhoun H, Levac D, et al. PRISMA extension for scoping reviews (PRISMA-ScR): checklist and explanation. Ann Intern Med. 2018;169(7):467-73. doi: 10.7326/m18-0850.

11. Peters MDJ, Marnie C, Tricco AC, Pollock D, Munn Z, Alexander L, et al. Updated methodological guidance for the conduct of scoping reviews. JBI Evid Implementation. 2021;19(1):3-10. doi: 10.1097/xeb.0000000000000277.
