# Supplementary material for: The global impact of COVID-19 on tuberculosis: A thematic scoping review, 2020–2023
Source: PLOS Glob Public Health. 2024 Jul 3;4(7):e0003043. doi: 10.1371/journal.pgph.0003043 (PMC11221697; doi:10.1371/journal.pgph.0003043)
Supplement: S2 Text — (DOCX) [file pgph.0003043.s002.docx]

**S2 Text: Search Terms for Citation Selection**

**1. PubMed**

(( Mycobacterium tuberculosis or tuberculosis or TB [MeSH Terms] )) AND (( COVID-19 or COVID or pandemic COVID-19 or coronavirus or sars-cov-2 [MeSH Terms] )) AND ((primary healthcare or health services or healthcare system [MeSH Terms]) ))

*Used “MeSH Major Topics,” Dates: 01/01/2020 - 04/30/2023 & English language only*

**2. EBSCO**

( “Mycobacterium tuberculosis” or tuberculosis or TB ) and ( COVID-19 or COVID or “pandemic COVID-19” or coronavirus or sars-cov-2) and ( “primary healthcare” or “health services” or “healthcare system”)

*Used “Boolean,” the default search mode in EBSCO, 01/01/2020 - 04/30/2023 & English language only*

**3. Google Scholar**

“Mycobacterium tuberculosis”|tuberculosis|TB COVID-19|COVID|“pandemic COVID-19”| |coronavirus|sars-cov-2 “primary healthcare”|“health services”|“healthcare system”

*Used “Keywords,” Dates: 01/01/2020 - 04/30/2023 & English language only*
