# Supplementary material for: The global impact of COVID-19 on tuberculosis: A thematic scoping review, 2020–2023
Source: PLOS Glob Public Health. 2024 Jul 3;4(7):e0003043. doi: 10.1371/journal.pgph.0003043 (PMC11221697; doi:10.1371/journal.pgph.0003043)
Supplement: S1 Table — (DOCX) [file pgph.0003043.s003.docx]

**S1 Table: Data Extraction Instrument for Thematic Characterization**

Name: _________________________

| **Author** | **Year** | **Country/ Region/ Continent/Global** | **Type of Publication** | **Theme: 1^st^ choice** | **Themes: 2^nd^ and 3^rd^ choice based on a publication’s discussion**  **of a specific cause, effect, or substantive recommendation**  **germane to another theme** | |
| --- | --- | --- | --- | --- | --- | --- |
|  |  |  |  |  | **2^nd^ choice** | **3^rd^ choice** |
|  |  |  |  |  |  |  |
|  |  |  |  |  |  |  |
|  |  |  |  |  |  |  |
|  |  |  |  |  |  |  |
|  |  |  |  |  |  |  |
|  |  |  |  |  |  |  |
|  |  |  |  |  |  |  |
|  |  |  |  |  |  |  |
|  |  |  |  |  |  |  |
|  |  |  |  |  |  |  |
|  |  |  |  |  |  |  |
|  |  |  |  |  |  |  |
|  |  |  |  |  |  |  |
|  |  |  |  |  |  |  |
|  |  |  |  |  |  |  |
