# Supplementary material for: The global impact of COVID-19 on tuberculosis: A thematic scoping review, 2020–2023
Source: PLOS Glob Public Health. 2024 Jul 3;4(7):e0003043. doi: 10.1371/journal.pgph.0003043 (PMC11221697; doi:10.1371/journal.pgph.0003043)
Supplement: S3 Table — (DOCX) [file pgph.0003043.s005.docx]

**S3 Table: Thematic Categorization of Publication* [n=176]**

**Case Finding and Notification [n=45; 26%]**

| **Author** | **Year of Publication** | **Country/Region/ Continent/Global** | **Type of Publication/Research^†, ‡^** |
| --- | --- | --- | --- |
| Alene, et al. [1] | 2020 | Australia | Editorial |
| Arentz, et al. [2] | 2022 | India | OR^§^ [Modelling] |
| Aznar, et al. [3] | 2022 | Spain | OR [Survey] |
| Bhargava, et al. [4] | 2020 | India | Editorial |
| Burzynski, et al. [5] | 2020 | United States | Letter |
| Chan, et al. [6] | 2021 | Indonesia | Editorial |
| Choi, et al. [7] | 2021 | South Korea | OR [Cohort] |
| Cilloni, et al. [8] | 2020 | Global | OR [Modelling] |
| Crowder, et al. [9] | 2021 | Philippines | OR [Modelling] |
| Datta, et al. [10] | 2020 | India | OR [Case finding] |
| De Souza, et al. [11] | 2020 | Brazil | Letter |
| Ding, et al. [12] | 2021 | China | OR [Modelling] |
| Fei, et al. [13] | 2020 | China | OR [Surveillance] |
| Fernandes Maia, et al. [14] | 2022 | Brazil | Letter |
| Gigante. et al. [15] | 2021 | Portugal | Letter |
| Godoy, et al. [16] | 2022 | Spain | OR [Surveillance] |
| Golandaj, JA [17] | 2022 | India | OR [Surveillance [PEDS]] |
| Hasan, et al. [18] | 2022 | Vietnam | OR [Cohort] |
| Iyengar, et al. [19] | 2020 | India | Editorial |
| Kadota, et al. [20] | 2020 | Uganda | Letter |
| Komiya, et al. [21] | 2020 | Japan | Letter |
| Kwak, et al. [22] | 2020 | South Korea | OR [Surveillance] |
| Lebina, et al. [23] | 2020 | South Africa | Letter |
| Lewer, et al. [24] | 2020 | United Kingdom | Editorial |
| Liu, et al. [25] | 2021 | China | OR [Surveillance] |
| Martin-Hughes, et al. [26] | 2022 | Global | OR [Modelling] |
| McQuaid, et al. [27] | 2022 | Global | OR [Modelling] |
| McQuaid, et al. [28] | 2020 | Global | Letter |
| Migliori, et al. [29] | 2022 | Global | OR [Surveillance] |
| Migliori, et al. [30] | 2021 | Global | Letter |
| Odume, et al. [31] | 2020 | Nigeria | OR [Surveillance] |
| Ortiz-Martinez, et al. [32] | 2022 | Columbia | Letter |
| Ozdemir, et al. [33] | 2022 | Turkey | OR [Surveillance] |
| Pai, et al. [34] | 2022 | Global | Editorial |
| Ranasinghe, et. al. [35] | 2022 | Global | OR [Modelling [PEDS]] |
| Ruhwald, et al. [36] | 2021 | Global | Editorial |
| Sahu, et al. [37] | 2021 | Global | Review |
| Tobing, et al. [38] | 2023 | Indonesia | OR [Surveillance] |
| Tok, et al. [39] | 2022 | Malaysia | Letter |
| Wang, et al. [40] | 2021 | China | OR [Surveillance] |
| WHO [41] | 2021 | Global | Review |
| Wu, et al. [42] | 2020 | China | OR [Cross-sectional] |
| Xu, et al. [43] | 2021 | China | OR [Modelling] |
| Xu, et al. [44] | 2023 | China | Letter |
| Zamani, et al. [45] | 2021 | Iran | Letter |

*Categorizing a publication’s theme often refers to a discussion of a specific cause, effect, or recommendation germane to another theme.

^†^A publication's categorization of an editorial, commentary, opinion, perspective, or reflection is titled “editorial.”

^‡^A publication's categorization of a letter to the editor, research letter, correspondence, or communication is titled “letter.”

^§^ OR: Original research

**Diagnosis and Laboratory Systems [n=19; 10.7%]**

| **Author** | **Year of Publication** | **Country/Region/ Continent/Global** | **Type of Publication/Research** |
| --- | --- | --- | --- |
| Awasthi, et al. [46] | 2021 | India | Letter |
| Bardhan, et al. [47] | 2021 | India | Letter |
| Di Gennaro, et al. [48] | 2021 | Italy | OR [Cohort] |
| Diallo, et al. [49] | 2022 | Burkina Faso | OR [Cross-sectional] |
| Hazra, et al. [50] | 2021 | India | OR [Cohort] |
| Lakoh, et al. [51] | 2021 | Sierra Leone | OR [Cohort] |
| Lestari, et al. [52] | 2022 | Indonesia | OR [Cohort] |
| Loh, et al. [53] | 2022 | Global | Editorial |
| MacLean, et al. [54] | 2022 | Global | Editorial |
| Malik, et al. [55] | 2020 | Pakistan | Editorial |
| Maurer, et al. [56] | 2021 | Europe | OR [Cohort] |
| Mohammed, et al. [57] | 2020 | Ethiopia | Editorial |
| Muñiz-Salazar, et al. [58] | 2022 | Mexico | OR [Mixed methods] |
| Nalunjogi, et al. [59] | 2023 | Global | OR [Survey] |
| Narita, et al. [60] | 2021 | United States | OR [case series] |
| Nikolayevskyy, et al. [61] | 2021 | Europe | Letter |
| Santos, et al. [62] | 2023 | Brazil | OR [Cross-sectional] |
| Schiza, et al. [63] | 2022 | United Kingdom | Letter |
| Tovar, et al. [64] | 2022 | Global | OR [Modelling] |

**Prevention, Treatment, and Care [n=22; 12.2%]**

| **Author** | **Year of Publication** | **Country/Region/ Continent/Global** | **Type of Publication/Research** |
| --- | --- | --- | --- |
| Abikoye, TM [65] | 2020 | Nigeria | OR [Case report] |
| Apolisi, et al. [66] | 2022 | South Africa | Editorial |
| Arega, et al. [67] | 2022 | Ethiopia | OR [Cohort] |
| Benade, et al. [68] | 2022 | South Africa | OR [Retrospective data analysis] |
| Caren, et al. [69] | 2022 | Indonesia | Review |
| Chilot, et al. [70] | 2021 | Ethiopia | OR [Cross-sectional] |
| Coutinho, et al. [71] | 2023 | Brazil | OR [Cohort] |
| Gandhi, et al. [72] | 2022 | India | OR [Cohort] |
| Geric, et al. [73] | 2022 | Canada | OR [Cohort] |
| Jeong, et al. [74] | 2023 | Global | OR [Rapid review] |
| Keshavjee, S. [75] | 2022 | Global | Proceedings |
| Louie, et al. [76] | 2021 | United States | OR [Cohort] |
| Magro, et al. [77] | 2020 | Italy | Letter |
| Meneguim, et al. [78] | 2020 | India | Letter |
| Min, et al. [79] | 2022 | South Korea | OR [Cross-sectional] |
| Mohr-Holland, et al. [80] | 2021 | South Africa | Editorial |
| Namkoong, et al. [81] | 2020 | Global | Letter |
| Padmapriyadarsini, et al. [82] | 2021 | Global | Editorial |
| Rai, et al. [83] | 2020 | India | Letter |
| Sahu, et al. [84] | 2022 | Global | Editorial |
| Shaikh, et al. [85] | 2021 | Global | OR [Modelling] |
| Tiberi, et al. [86] | 2021 | Global | Review |

**Telemedicine/Telehealth [n=12; 6.8%]**

| **Author** | **Year of Publication** | **Country/Region/ Continent/Global** | **Type of Publication/Research** |
| --- | --- | --- | --- |
| Bachina, et al. [87] | 2022 | United States | OR [Cohort] |
| Bhaita, et al. [88] | 2020 | Southeast Asia Region | Editorial |
| Calnan, et al. [89] | 2022 | Philippines | OR [Case-finding] |
| Cardensa-Escalante, et. al. [90] | 2022 | Peru | Letter |
| Fekadu, et al. [91] | 2021 | Global | OR [Cost-effectiveness] |
| Klinton, et al. [92] | 2021 | Global | Review |
| Koura, et al. [93] | 2020 | Africa | Letter |
| Kumwichar, et al. [94] | 2022 | Thailand | OR [Feasibility study] |
| Lippincott, et al. [95] | 2022 | United States | OR [Cohort] |
| Migliori, et al. [96] | 2020 | Global | OR [Survey] |
| Visca, et al. [97] | 2020 | Global | Editorial |
| Watts, et al. [98] | 2020 | Australia | Letter |

**Social Determinants of Health [n=14; 8%]**

| **Author** | **Year of Publication** | **Country/Region/ Continent/Global** | **Type of Publication/Research** |
| --- | --- | --- | --- |
| Bhargava & Shewade [99] | 2020 | India | Review |
| Chatterjee, et al. [100] | 2022 | India | OR [Cohort] |
| George, et al. [101] | 2022 | India | OR [Survey] |
| Khan, et al. [102] | 2021 | Peru | Letter |
| Knipper, et al. [103] | 2021 | Global | Review |
| Louie, et al. [104] | 2020 | United States | Letter |
| McQuaid, et al. [105] | 2021 | Global | Review |
| Ryckman, et al. [106] | 2023 | Global | Editorial |
| Saunders, et al. [107] | 2020 | Global | Editorial |
| Shrinivasan, et al. [108] | 2020 | India | Editorial |
| Singh, PK. [109] | 2022 | Global | Editorial |
| Souza, et al. [110] | 2021 | Brazil | OR [Survey] |
| Vanleeuw, et al. [111] | 2022 | South Africa | OR [Survey] |
| Zumla, et al. [112] | 2021 | Global | Editorial |

**Airborne Infection Prevention and Control [n=8; 4.6%]**

| **Author** | **Year of Publication** | **Country/Region/ Continent/Global** | **Type of Publication/Research** |
| --- | --- | --- | --- |
| Awan, et al. [113] | 2022 | Pakistan | Editorial |
| Cox, et al. [114] | 2020 | Global | Letter |
| Driessche, et al. [115] | 2021 | South Africa | Editorial |
| Jain, et al. [116] | 2020 | India | Review |
| Jamal, et al. [117] | 2020 | Pakistan | Letter |
| Mannan, et al. [118] | 2022 | India | OR [Survey] |
| Marti, et al. [119] | 2022 | Global | OR [Survey] |
| Masina, et al. [120] | 2022 | Eswatini | OR [Cohort] |

**Health System Strengthening [n=22; 13%]**

| **Author** | **Year of Publication** | **Country/Region/ Continent/Global** | **Type of Publication/Research** |
| --- | --- | --- | --- |
| Andom, et al. [121] | 2023 | Lesotho | OR [Modelling] |
| Berra, et al. [122] | 2022 | Brazil | OR [Ecological] |
| Bouaddi, et al. [123] | 2021 | Morocco | Letter |
| Chiang, et al. [124] | 2020 | Western Pacific Region | Letter |
| Cronin, et al. [125] | 2020 | United States | OR [Surveillance] |
| Dlangalala, et al. [126] | 2021 | Global | Review |
| Fuady, et al. [127] | 2020 | Global | Review |
| Khan, et al. [128] | 2022 | Pakistan | Review |
| Khobragade, et al. [129] | 2021 | India | Review |
| Kim, et al. [130] | 2022 | South Korea | OR [Modelling] |
| Klinton, et al. [131] | 2020 | Global | Review |
| Manhiça, et al. [132] | 2022 | Mozambique | OR [Modelling] |
| Mihika, et al. [133] | 2022 | Bangladesh | OR [Cross-sectional] |
| Min, et al. [134] | 2020 | South Korea | OR [Cohort] |
| Nhari, et al. [135] | 2020 | Zimbabwe | Letter |
| Oga-Omenka, et al. [136] | 2023 | Nigeria | OR [Survey] |
| Petersen, et al. [137] | 2022 | Global | Editorial |
| Reid, et al. [138] | 2020 | Global | Editorial |
| Roberts, et al. [139] | 2021 | Global | Editorial |
| Tran, et al. [140] | 2021 | Zambia | Editorial |
| van Rensburg, et al. [141] | 2022 | South Africa | Review |
| Yadav, et al. [142] | 2021 | India | Editorial |

**Mental Health [n=13; 7.4%]**

| **Author** | **Year of Publication** | **Country/Region/ Continent/Global** | **Type of Publication/Research** |
| --- | --- | --- | --- |
| ACTION, et al. [143] | 2020 | Global | OR [Survey] |
| Ferrer, et al. [144] | 2021 | Philippines | Editorial |
| Loveday, et al. [145] | 2020 | Global | Review |
| Malik, et al. [146] | 2022 | Pakistan | OR [Mixed methods] |
| Mandal, et al. [147] | 2022 | India | Original research |
| Millones, et al. [148] | 2022 | Peru | OR [Mixed methods] |
| Mwamba, et al. [149] | 2020 | Zambia | OR [Survey] |
| Santos, et al. [150] | 2021 | Brazil | OR [Survey] |
| Sunjaya, et al. [151] | 2022 | India | OR [Cross-sectional] |
| Tinoco, et al. [152] | 2022 | Portugal | Letter |
| Togun, et al. [153] | 2020 | Global | Review |
| Zhang, et al. [154] | 2022 | China | OR [Survey] |
| Zimmer, et al. [155] | 2021 | Global | OR [Survey] |

**Stigma [n=11; 6.3%]**

| **Author** | **Year of Publication** | **Country/Region/ Continent/Global** | **Type of Publication/Research** |
| --- | --- | --- | --- |
| Beyene, et al. [156] | 2022 | Iran | OR [Cohort] |
| Buonsenso, et al. [157] | 2021 | Sierra Leone | Letter |
| Dheda, et al. [158] | 2022 | Global | Review |
| Fatima, et al. [159] | 2021 | Global | Review |
| Islam, et al. [160] | 2020 | Pakistan | Letter |
| Kumar, et al. [161] | 2020 | Global | Letter |
| Maroof, et al. [162] | 2022 | India | OR [Survey] |
| Oga-Omenka, et al. [163] | 2021 | Global | Editorial |
| Soko, et al. [164] | 2021 | Malawi | OR [Surveillance] |
| Uwishema, et al. [165] | 2022 | Africa | Editorial |
| Zimmer, et al. [166] | 2022 | Global | Editorial |

**Health Education (n=10; 5.7%]**

| **Author** | **Year of Publication** | **Country/Region/ Continent/Global** | **Type of Publication/Reasearch** |
| --- | --- | --- | --- |
| Anigbo, et al. [167] | 2022 | Nigeria | Review |
| Chapman, et al. [168] | 2021 | Global | Review |
| Chen & Zhang [169] | 2020 | China | Letter |
| Lipman, et al. [170] | 2021 | Global | Editorial |
| Lungu, et al. [171] | 2022 | Zambia | OR [Case-finding] |
| Nkereuwem, et al. [172] | 2021 | Global | OR [Survey] |
| Rodrigues, et al. [173] | 2022 | Portugal | OR [Survey] |
| `Shahnavazi, et al. [174] | 2022 | Iran | OR [Mixed methods] |
| Tale & Soibam [175] | 2021 | Global | Editorial |
| Wingfield, et al. [176] | 2021 | Global | Editorial |

**References**

1. Alene KA, Wangdi K, Clements ACA. Impact of the COVID-19 pandemic on tuberculosis control: An Overview. Trop Med Infect Dis. 2020;5(3):123. doi: 10.3390/tropicalmed5030123.

2. Arentz M, Ma J, Zheng P, Vos T, Murray CJL, Kyu HH**.** The impact of the COVID-19 pandemic and associated suppression measures on the burden of tuberculosis in India. BMC Infect Dis. 2022;22(1):92. doi: 10.1186/s12879-022-07078-y.

3. Aznar ML, Espinosa-Pereiro J, Saborit N, Jové N, Sánchez Martinez F, Pérez-Recio S, et al. Impact of the COVID-19 pandemic on tuberculosis management in Spain. Int J Infect Dis. 2021;108:300-5. doi: 10.1016/j.ijid.2021.04.075.

4. Bhargava A, Bhargava M, Meher A**.** Universal health coverage and tuberculosis care in India in the times of Covid-19: Aligning Ayushman Bharat (National Health Assurance Scheme) to improve case detection, reduce deaths and catastrophic health expenditure. Natl Med J India. 2020;33(5):298-301. doi: 10.4103/0970-258x.303111.

5. Burzynski J, Macaraig M, Nilsen D, Schluger NW**.** Transforming essential services for tuberculosis during the COVID-19 pandemic: lessons from New York City. Int J Tuberc Lung Dis. 2020;24(7):735-6. doi: 10.5588/ijtld.20.0283.

6. Chan G, Triasih R, Nababan B, du Cros P, Wilks N, Main S, et al. Adapting active case-finding for TB during the COVID-19 pandemic in Yogyakarta, Indonesia. Public Health Action. 2021;11(2):41-9. doi: 10.5588/pha.20.0071.

7. Choi H, Ko Y, Lee CY, Chung SJ, Kim HI, Kim JH, et al. Impact of COVID-19 on TB epidemiology in South Korea. Int J Tuberc Lung Dis. 2021;25(10):854-60. doi: 10.5588/ijtld.21.0255.

8. Cilloni L, Fu H, Vesga JF, Dowdy D, Pretorius C, Ahmedov S, et al. The potential impact of the COVID-19 pandemic on the tuberculosis epidemic a modelling analysis. EClinicalMedicine. 2020;28:100603. doi: 10.1016/j.eclinm.2020.100603

9. Crowder R, Geocaniga-Gaviola DM, Fabella RA, Lim A, Lopez E, Kadota JL, et al. Impact of shelter-in-place orders on TB case notifications and mortality in the Philippines during the COVID-19 pandemic. J Clin Tuberc Other Mycobact Dis. 2021;25:100282. doi: 10.1016/j.jctube.2021.100282.

10. Datta B, Jaiswal A, Goyal P, Prakash A, Tripathy JP, Trehan N**.** The untimely demise of the TB Free block model in the wake of coronavirus disease 2019 in India. Trans R Soc Trop Med Hyg. 2020;114(11):789-91. doi: 10.1093/trstmh/traa067.

11. de Souza CDF, Coutinho HS, Costa MM, Magalhaes M, Carmo RF**.** Impact of COVID-19 on TB diagnosis in Northeastern Brazil. Int J Tuberc Lung Dis. 2020;24(11):1220-2. doi: 10.5588/ijtld.20.0661.

12. Ding W, Li Y, Bai Y, Li Y, Wang L, Wang Y**.** Estimating the effects of the COVID-19 outbreak on the reductions in tuberculosis cases and the epidemiological trends in China: a causal impact analysis. Infect Drug Resist. 2021;14:4641-55. doi: 10.2147/IDR.S337473.

13. Fei H, Yinyin X, Hui C, Ni W, Xin D, Wei C, et al. The impact of the COVID-19 epidemic on tuberculosis control in China. Lancet Reg Health West Pac. 2020;3:100032. doi: 10.1016/j.lanwpc.2020.100032.

14. Maia CMF, Martelli DRB, Silveira D, Oliveira EA, Martelli Junior H**.** Tuberculosis in Brazil: the impact of the COVID-19 pandemic. J Bras Pneumol. 2022;48(2):e20220082. doi: 10.36416/1806-3756/e20220082.

15. Gigante AR, Sousa M, Aguiar A, Pinto M, Gaio R, Duarte R**.** The impact of COVID-19 on the TB response: data from the field. Int J Tuberc Lung Dis. 2021;25(9):769-71. doi: 10.5588/ijtld.21.0149.

16. Godoy P, Parrón I, Barrabeig I, Caylà JA, Clotet L, Follia N, et al. Impact of the COVID-19 pandemic on contact tracing of patients with pulmonary tuberculosis. European Journal of Public Health. 2022;32(4):643-7. doi: 10.1093/eurpub/ckac031.

17. Golandaj JA. Pediatric TB detection in the era of COVID-19. Indian J Tuberc. 2022;69(1):104-8. doi: 10.1016/j.ijtb.2021.04.015.18.

18. Hasan T, Nguyen VN, Nguyen HB, Nguyen TA, Le HTT, Pham CD, et al. Retrospective Cohort Study of Effects of the COVID-19 Pandemic on Tuberculosis Notifications, Vietnam, 2020. Emerg Infect Dis. 2022;28(3):684-92. doi: 10.3201/eid2803.211919.

19. Iyengar KP, Jain VK**.** Tuberculosis and COVID-19 in India- double trouble! Indian J Tuberc. 2020;67(4s):S175-6. doi: 10.1016/j.ijtb.2020.07.014.

20. Kadota JL, Reza TF, Nalugwa T, Kityamuwesi A, Nanyunja G, Kiwanuka N, et al. Impact of shelter-in-place on TB case notifications and mortality during the COVID-19 pandemic. Int J Tuberc Lung Dis. 2020;24(11):1212-14. doi: 10.5588/ijtld.20.0626.

21. Komiya K, Yamasue M, Takahashi O. The COVID-19 pandemic and the true incidence of Tuberculosis in Japan. J Infect. 2020;81(3):e24-e25. doi: 10.1016/j.jinf.2020.07.004.

22. Kwak N, Hwang S-S, Yim J-J**.** Effect of COVID-19 on tuberculosis notification, South Korea. Emerg Infect Dis. 2020;26(10):2506-8. doi: 10.3201/eid2610.202782.

23. Lebina L, Dube M, Hlongwane K, Brahmbatt H, Lala SG, Reubenson G, et al. Trends in paediatric tuberculosis diagnoses in two South African hospitals early in the COVID-19 pandemic. S. Afr. Med. J.. 2020;110(12):1149-51. doi: 10.7196/SAMJ.2020.v110i12.15386.

24. Lewer D, Mulchandani R, Roche A, Cosgrove C, Anderson C**.** Why has the incidence of tuberculosis not reduced in London during the COVID-19 pandemic? Lancet Respir Med. 2022;10(3):231-3. doi: 10.1016/s2213-2600(22)00012-1.

25. Liu Q, Lu P, Shen Y, Li C, Wang J, Zhu L**.** Collateral impact of the coronavirus disease 2019 (COVID-19) pandemic on tuberculosis control in Jiangsu Province, China. Clin Infecti Dis. 2021;73(3):542-4. doi: 10.1093/cid/ciaa1289.

26. Martin-Hughes R, Vu L, Cheikh N, Kelly SL, Fraser-Hurt N, Shubber Z, et al. Impacts of COVID-19-related service disruptions on TB incidence and deaths in Indonesia, Kyrgyzstan, Malawi, Mozambique, and Peru: implications for national TB responses. PLOS Glob Public Health. 2022;2(3):e0000219. doi: 10.1371/journal.pgph.0000219.

27. McQuaid CF, Henrion MYR, Burke RM, MacPherson P, Nzawa-Soko R, Horton KC**.** Inequalities in the impact of COVID-19-associated disruptions on tuberculosis diagnosis by age and sex in 45 high TB burden countries. BMC Med. 2022;20(1):432. doi: 10.1186/s12916-022-02624-6.

28. McQuaid CF, McCreesh N, Read JM, Sumner T, Houben R, White RG, et al. The potential impact of COVID-19-related disruption on tuberculosis burden. Eur Respir J. 2020;56(2):2001718. doi: 10.1183/13993003.01718-2020.

29. Migliori GB, Thong PM, Alffenaar JW**.** Country-specific lockdown measures in response to the COVID-19 pandemic and its impact on tuberculosis control: a global study. J Bras Pneumol. 2022;48(2):e20220087. doi: 10.36416/1806-3756/e20220087.

30. Migliori GB, Thong PM, Alffenaar JW, Denholm J, Tadolini M, Alyaquobi F, et al. Gauging the impact of the COVID-19 pandemic on tuberculosis services: a global study. Eur Respir J. 2021;58(5) doi: 10.1183/13993003.01786-2021.

31. Odume B, Falokun V, Chukwuogo O, Ogbudebe C, Useni S, Nwokoye N, et al. Impact of COVID-19 on TB active case finding in Nigeria. Public Health Action. 2020;10(4):157-62. doi: 10.5588/pha.20.0037.

32. Ortiz-Martínez Y, Rodríguez-Morales AJ, Henao-Martínez AF**.** Decreased notification of TB cases during the COVID-19 pandemic. Int J Tuberc Lung Dis. 2022;26(2):177-8. doi: 10.5588/ijtld.21.0651.

33. Ozdemir S, Oztomurcuk D, Oruc MA**.** Impact of the COVID-19 pandemic on tuberculosis patients and tuberculosis control programs in Turkey, review and analysis. Arch Public Health. 2022;80(1):252. doi: 10.1186/s13690-022-01007-w.

34. Pai M, Kasaeva T, Swaminathan S**.** Covid-19’s devastating effect on tuberculosis care—a path to recovery. N Engl J Med. 2022;386(16):1490-3. doi: 10.1056/NEJMp2118145.

35. Ranasinghe L, Achar J, Gröschel MI, Whittaker E, Dodd PJ, Seddon JA**.** Global impact of COVID-19 on childhood tuberculosis: an analysis of notification data. Lancet Glob Health. 2022;10(12):e1774-81. doi: 10.1016/s2214-109x(22)00414-4.

36. Ruhwald M, Carmona S, Pai M**.** Learning from COVID-19 to reimagine tuberculosis diagnosis. The Lancet Microbe. 2021;2(5):e169-70. doi: 10.1016/s2666-5247(21)00057-4.

37. Sahu S, Ditiu L, Sachdeva KS, Zumla A**.** Recovering from the Impact of the Covid-19 Pandemic and Accelerating to Achieving the United Nations General Assembly Tuberculosis Targets. Int J Infect Dis. 2021;113 Suppl 1:S100-3. doi: 10.1016/j.ijid.2021.02.078.

38. Tobing KL, Letelay AM, Senewe FP, Lolong DB, Pracoyo NE, Susanti L, et al. TB Active case-finding before and during the Covid-19 pandemic. Proceedings of the 1st International Conference for Health Research – BRIN (ICHR 2022); 2023:469-81.;2023:469-81.

39. Tok PSK, Kamarudin NA, Jamaludin M, Ab Razak MF, Ahmad MAS, Abu Bakar FA, et al. Effect of COVID-19 on tuberculosis notification in Johor Bahru, Malaysia. Infect Dis. 2022;54(3):235-7. doi: 10.1080/23744235.2021.2000636.

40. Wang X, He W, Lei J, Liu G, Huang F, Zhao Y**.** Impact of COVID-19 pandemic on pre-treatment delays, detection, and clinical characteristics of tuberculosis patients in Ningxia Hui Autonomous Region, China. Front Public Health. 2021;9:644536. doi: 10.3389/fpubh.2021.644536.

41. World Health Organization. Impact of the COVID-19 pandemic on TB detection and mortality in 2020. Available from: [https://www.who.int/publications/m/item/impact-of-the-covid-19-pandemic-on-tb-detection-and-mortality-in-2020 on May 9, 2023](file:////Volumes/SanDisk512/C.%20%20%20%20on.%20%20%20%20T./DRAFTS%20OF%20LIT%20REVIEW/V2%20MOST%20RECENT%20/11th%20HOUR/EDITING/KEN'S/World%20Health%20Organization.%20Impact%20of%20the%20COVID-19%20pandemic%20on%20TB%20detection%20and%20mortality%20in%202020.%20Accessed%20at:%20https:/www.who.int/publications/m/item/impact-of-the-covid-19-pandemic-on-tb-detection-and-mortality-in-2020%20on%20May%209,%202023)

42. Wu Z, Chen J, Xia Z, Pan Q, Yuan Z, Zhang W, et al. Impact of the COVID-19 pandemic on the detection of TB in Shanghai, China. Int J Tuberc Lung Dis. 2020;24(10):1122-4. doi: 10.5588/ijtld.20.0539.

43. Xu C, Li T, Hu D, Zhang H, Zhao Y, Liu J**.** Predicted Impact of the COVID-19 responses on deaths of tuberculosis - China, 2020. China CDC Wkly. 2021;3(2):21-4. doi: 10.46234/ccdcw2021.004.

44. Xu J, Wang Y, Liu F, Yang H**.** Changes of tuberculosis infection in mainland China before and after the COVID-19 pandemic. J Infect. 2023;86(2):154-225. doi: 10.1016/j.jinf.2022.12.008.

45. Zamani S, Honarvar MR, Behnampour N, Sheikhy M, Sedaghat M, Ghaemi S, et al. Decline in TB incidence during the COVID-19 pandemic. Int J Tuberc Lung Dis. 2021;25(12):1043-4. doi: 10.5588/ijtld.21.0548.

46. Awasthi AK, Singh PK**.** Tuberculosis management in India during COVID-19 crisis. J Public Health Policy. 2021;42(1):185-9. doi: 10.1057/s41271-020-00265-8..

47. Bardhan M, Hasan MM, Ray I, Sarkar A, Chahal P, Rackimuthu S, et al. Tuberculosis amidst COVID-19 pandemic in India: unspoken challenges and the way forward. Trop Med Health. 2021;49(1):84. doi: 10.1186/s41182-021-00377-1.

48. Di Gennaro F, Gualano G, Timelli L, Vittozzi P, Di Bari V, Libertone R, et al. Increase in tuberculosis diagnostic delay during first wave of the COVID-19 pandemic: data from an Italian infectious disease referral hospital. Antibiotics (Basel). 2021;10(3):272. doi: 10.3390/antibiotics10030272..

49. Diallo A, Combary A, Veronese V, Dahourou DL, Ouédraogo S, Traoré IT, et al. Delays in TB diagnosis and treatment initiation in Burkina Faso during the COVID-19 pandemic. Trop Med Infect Dis. 2022;7(9):232. doi: 10.3390/tropicalmed7090237.

50. Hazra D, Chawla K, Shenoy VP, Pandey AK, S N**.** The aftermath of COVID-19 pandemic on the diagnosis of TB at a tertiary care hospital in India. J Infect Public Health. 2021;14(8):1095-8. doi: 10.1016/j.jiph.2021.07.001.

51. Lakoh S, Jiba DF, Baldeh M, Adekanmbi O, Barrie U, Seisay AL, et al. Impact of COVID-19 on tuberculosis case detection and treatment outcomes in Sierra Leone. Trop Med Infect Dis. 2021;6(3):154. doi: 10.3390/tropicalmed6030154.

52. Lestari T, Kamaludin, Lowbridge C, Kenangalem E, Poespoprodjo JR, Graham SM, et al. Impacts of tuberculosis services strengthening and the COVID-19 pandemic on case detection and treatment outcomes in Mimika District, Papua, Indonesia: 2014-2021. PLOS Glob Public Health. 2022;2(9):e0001114. doi: 10.1371/journal.pgph.0001114.

53. Loh FK, Thong PM, Ong CWM**.** The crucial need for tuberculosis translational research in the time of COVID-19. Lancet Respir Med. 2022;10(6):531-3. doi: 10.1016/s2213-2600(22)00099-6.

54. MacLean EL, Villa-Castillo L, Ruhwald M, Ugarte-Gil C, Pai M**.** Integrated testing for TB and COVID-19. Med (N Y). 2022;3(3):162-6. doi: 10.1016/j.medj.2022.02.002.

55. Malik AA, Safdar N, Chandir S, Khan U**.** Tuberculosis control and care in the era of COVID-19. Health Policy Plan. 2020;35(8):1130-32. doi: 10.1093/heapol/czaa109.

56. Maurer FP, Shubladze N, Kalmambetova G, Felker I, Kuchukhidze G, Drobniewski F, et al. Impact of the COVID-19 pandemic on tuberculosis national reference laboratory services in the WHO European Region, March to November 2020. Euro Surveill. 2021;26(24):2100426. doi: 10.2807/1560-7917.Es.2021.26.24.2100426.

57. Mohammed H, Oljira L, Roba KT, Yimer G, Fekadu A, Manyazewal T**.** Containment of COVID-19 in Ethiopia and implications for tuberculosis care and research. Infect Dis Poverty. 2020;9(1):131. doi: 10.1186/s40249-020-00753-9.

58. Muñiz-Salazar R, Le T, Cuevas-Mota J, González-Fagoaga JE, Zapata-Garibay R, Ruiz-Tamayo PS, et al. Impact of COVID-19 on tuberculosis detection and treatment in Baja California, México. Front Public Health. 2022;10:921596. doi: 10.3389/fpubh.2022.921596.

59. Nalunjogi J, Mucching-Toscano S, Sibomana JP, Centis R, D'Ambrosio L, Alffenaar JW, et al. Impact of COVID-19 on diagnosis of tuberculosis, multidrug-resistant tuberculosis, and on mortality in 11 countries in Europe, Northern America, and Australia. A Global Tuberculosis Network study. Int J Infect Dis. Int J Infect Dis. 2023;130(Suppl 1):S25-9. doi: 10.1016/j.ijid.2023.02.025.

60. Narita M, Hatt G, Toren KG**.** Narita M, Hatt G, Toren KG**.** Delayed tuberculosis diagnoses during the coronavirus disease 2019 (COVID-19) pandemic in 2020—King County, Washington. Clin Infect Dis. 2021;73(Suppl 1):S74-6. doi: 10.1093/cid/ciab387.

61. Nikolayevskyy V, Holicka Y, van Soolingen D, van der Werf MJ, Ködmön C, Surkova E, et al. Impact of COVID-19 on tuberculosis case detection and treatment outcomes in Sierra Leone.Eur Respir J. 2021;57(1):154. doi: 10.1183/13993003.03890-2020.

62. Santos VS, Allgayer MF, Kontogianni K, Rocha JE, Pimentel BJ, Amorim MTP, et al. Pooling of sputum samples to increase tuberculosis diagnostic capacity in Brazil during the COVID-19 pandemic. Int J Infect Dis. 2023;129:10-4. doi: 10.1016/j.ijid.2023.01.009.

63. Schiza V, Kruse M, Xiao Y, Kar S, Lovejoy K, Wrighton-Smith P, et al. Impact of the COVID-19 pandemic on TB infection testing. Int J Tuberc Lung Dis. 2022;26(2):174-6. doi: 10.5588/ijtld.21.0628.

64. Tovar M, Aleta A, Sanz J, Moreno Y**.** Modelling the impact of COVID-19 on future tuberculosis burden. Commun Med (Lond). 2022;2:77. doi: 10.1038/s43856-022-00145-0.

65. Abikoye TM**.** Collateral damage: the impact of the COVID-19 pandemic on the care of a patient with tuberculous neuroretinitis in Lagos, Nigeria. Pan Afr Med J. 2020;35(Suppl 2):S135. doi: 10.11604/pamj.supp.2020.35.135.24691.

66. Apolisi I, Mema N, Tyeku N, Beko B, Memani B, Daniels J, et al. Supporting families with tuberculosis during COVID-19 in Khayelithsa, South Africa. Lancet Respir Med. 2022;10(6):542-3. doi: 10.1016/s2213-2600(22)00121-7.

67. Arega B, Negesso A, Taye B, Weldeyohhans G, Bewket B, Negussie T, et al. Impact of COVID-19 pandemic on TB prevention and care in Addis Ababa, Ethiopia: a retrospective database study. BMJ Open. 2022;12(2):e053290. doi: 10.1136/bmjopen-2021-053290.

68. Benade M, Long L, Meyer-Rath G, Miot J, Evans D, Tucker JM, et al. Reduction in initiations of drug-sensitive tuberculosis treatment in South Africa during the COVID-19 pandemic: analysis of retrospective, facility-level data. PLOS Glob Public Health. 2022;2(10):e0000559. doi: 10.1371/journal.pgph.0000559.

69. Caren GJ, Iskandar D, Pitaloka DAE, Abdulah R, Suwantika AA**.** COVID-19 pandemic disruption on the management of tuberculosis treatment in Indonesia. J Multidiscip Healthc. 2022;15:175-83. doi: 10.2147/jmdh.S341130.

70. Chilot D, Woldeamanuel Y, Manyazewal T**.** Real-time impact of COVID-19 on clinical care and treatment of patients with tuberculosis: a multicenter cross-sectional study in Addis Ababa, Ethiopia. Ann Glob Health. 2021;87(1):109. doi: 10.5334/aogh.3481.

71. Coutinho I, Alves LC, Werneck GL, Trajman A**.** The impact of the COVID-19 pandemic in tuberculosis preventive treatment in Brazil: a retrospective cohort study using secondary data. Lancet Reg Health Am. 2023;19:100444. doi: 10.1016/j.lana.2023.100444.

72. Gandhi AP, Kathirvel S, Rehman T**.** Effect of COVID-19 lockdown on the pathway of care and treatment outcome among patients with tuberculosis in a rural part of northern India: a community-based study. J Rural Med. 2022;17(2):59-66. doi: 10.2185/jrm.2021-039.

73. Geric C, Saroufim M, Landsman D, Richard J, Benedetti A, Batt J, et al. Impact of COVID-19 on tuberculosis prevention and treatment in Canada: a multicenter analysis of 10,833 patients. 2022;225(8):1317-20. doi: 10.1093/infdis/jiab608.

74. Jeong Y, Min J. Impact of COVID-19 pandemic on tuberculosis preventive services and their post-pandemic recovery strategies: a rapid review of literature. J Korean Med Sci. 2023;38(5):e43. doi: 10.3346/jkms.2023.38.e43.

75. Keshavjee S**.** Progress toward global tuberculosis elimination goals and opportunities for moving forward. In: Biffl C, Liao J, Nicholson A, eds. Innovations for tackling tuberculosis in the time of COVID-19: proceedings of a workshop, 2022. National Academies of Sciences, Engineering, and Medicine. The National Academies Press, Washington, DC. Available from: <https://nap.nationalacademies.org/catalog/26530/innovations-for-tackling-tuberculosis-in-the-time-of-covid-19>.

76. Louie JK, Agraz-Lara R, Romo L, Crespin F, Chen L, Graves S**.** Tuberculosis-associated hospitalizations and deaths after COVID-19 shelter-in-place, San Francisco, California, USA. Emerg Infect Dis. 2021;27(8):2227-9. doi: 10.3201/eid2708.210670.

77. Magro P, Formenti B, Marchese V. Impact of the SARS-CoV-2 epidemic on tuberculosis treatment outcome in Northern Italy. Eur Respir J. 2020;56(4):2002665. doi: 10.1183/13993003.02665-2020.

78. Meneguim AC, Rebello L, Das M, Ravi S, Mathur T, Mankar S, et al. Adapting TB services during the COVID-19 pandemic in Mumbai, India. Int J Tuberc Lung Dis. 2020;24(10):1119-21. doi: 10.5588/ijtld.20.0537.

79. Min J, Ko Y, Kim HW, Koo HK, Oh JY, Jeong YJ, et al. Increased healthcare delays in tuberculosis patients during the first wave of COVID-19 pandemic in Korea: a nationwide cross-sectional study. J Korean Med Sci. 2022;37(3):e20. doi: 10.3346/jkms.2022.37.e20.

80. Mohr-Holland E, Douglas-Jones B, Apolisi I**.** Tuberculosis preventive therapy for children and adolescents: an emergency response to the COVID-19 pandemic. Lancet Child Adolesc Health. 2021;5(3):159-61. doi: 10.1016/S2352-4642(21)00003-1.

81. Namkoong H, Horita N, Ebina-Shibuya R**.** Concern over a COVID-19-related BCG shortage. The International Journal of Tuberculosis and Lung Disease. 2020;24(6):642-3. doi: 10.5588/ijtld.20.0240.

82. Padmapriyadarsini C, Banurekha V, Arora VK**.** Challenges in TB control and the anticipated COVID-19 third wave: Way forward. Indian J Tuberc. 2021;68(4):425-7. doi: 10.1016/j.ijtb.2021.07.014.

83. Rai DK, Kumar R, Pandey SK**.** Problems faced by tuberculosis patients during COVID-19 pandemic: urgent need to intervene. Indian J Tuberc. 2020;67(4s):S173-4. doi: 10.1016/j.ijtb.2020.07.013.

84. Sahu S, Wandwalo E, Arinaminpathy N. Exploring the impact of the COVID-19 pandemic on tuberculosis care and prevention. J Pediatric Infect Dis Soc. 2022;11(Suppl 3):S67-71. doi: 10.1093/jpids/piac102.

85. Shaikh N, Pelzer PT, Thysen SM, Roy P, Harris RC, White RG**.** Impact of COVID-19 disruptions on global BCG coverage and paediatric TB mortality: a modelling study. Vaccines. 2021;9(11):1228. doi: 10.3390/vaccines9111228.

86. Tiberi S, Vjecha MJ, Zumla A, Galvin J, Migliori GB, Zumla A**.** Accelerating development of new shorter TB treatment regimens in anticipation of a resurgence of multi-drug resistant TB due to the COVID-19 pandemic. Int J Infect Dis. 2021;113(Suppl 1):S96-9. doi: 10.1016/j.ijid.2021.02.067.

87. Bachina P, Lippincott CK, Perry A, Munk E, Maltas G, Bohr R, et al. Programmatic adoption and implementation of video-observed therapy in Minnesota: prospective observational cohort study. JMIR Form Res. 2022;6(8):e38247. doi: 10.2196/38247.

88. Bhatia V, Mandal PP, Satyanarayana S, Aditama TY, Sharma M**.** Mitigating the impact of the COVID-19 pandemic on progress towards ending tuberculosis in the WHO South-East Asia Region. WHO South East Asia J Public Health. 2020;9(2):95-9. doi: 10.4103/2224-3151.294300.

89. Calnan M, Moran A, Jassim AlMossawi H**.** Maintaining essential tuberculosis services during the COVID-19 pandemic, Philippines. Bull World Health Organ. 2022;100(2):127-34. doi: 10.2471/blt.21.286807.

90. Cardenas-Escalante J, Fernandez-Saucedo J, Cubas WS**.** Impact of the COVID-19 pandemic on tuberculosis in Peru: Are we forgetting anyone? Enferm Infecc Microbiol Clin (Engl Ed). 2022;40(1):46-7. doi: 10.1016/j.eimce.2021.07.008.

91. Fekadu G, Jiang X, Yao J, You JHS**.** Cost-effectiveness of video-observed therapy for ambulatory management of active tuberculosis during the COVID-19 pandemic in a high-income country. Int J Infect Dis. 2021;113:271-8. doi: 10.1016/j.ijid.2021.10.029.

92. Klinton JS, Heitkamp P, Rashid A, Faleye BO, Win Htat H, Hussain H, et al. One year of COVID-19 and its impact on private provider engagement for TB: A rapid assessment of intermediary NGOs in seven high TB burden countries. J Clin Tuberc Other Mycobact Dis. 2021;25:100277. doi: 10.1016/j.jctube.2021.100277.

93. Koura KG, Harries AD, Fujiwara PI, Dlodlo RA, Sansan EK, Kampoer B, et al. COVID-19 in Africa: community and digital technologies for tuberculosis management. Int J Tuberc Lung Dis. 2020;24(8):863-5. doi: 10.5588/ijtld.20.0412.

94. Kumwichar P, Chongsuvivatwong V, Prappre T**.** Video-observed therapy with a notification system for improving the monitoring of tuberculosis treatment in Thailand: usability study. JMIR Form Res. 2022;6(5):e35994. doi: 10.2196/35994.

95. Lippincott CK, Perry A, Munk E, Maltas G, Shah M**.** Tuberculosis treatment adherence in the era of COVID-19. BMC Infect Dis. 2022;22(1):800. doi: 10.1186/s12879-022-07787-4.

96. Migliori GB, Thong PM, Akkerman O, Alffenaar JW, Álvarez-Navascués F, Assao-Neino MM, et al. Worldwide effects of coronavirus disease pandemic on tuberculosis services, January-April 2020. Emerg Infect Dis. 2020;26(11):2709-12. doi: 10.3201/eid2611.203163.

97. Visca D, Tiberi S, Pontali E, Spanevello A, Migliori, GB. Tuberculosis in the time of COVID-19: quality of life and digital innovation. Eur Respir J. 2020;56(2):2001998. doi: 10.1183/13993003.01998-2020.

98. Watts K, McKeown A, Denholm J, Baker AM**.** Responding to COVID-19: adjusting TB services in a low-burden setting. Int J Tuberc Lung Dis. 2020;24(8):866-9. doi: 10.5588/ijtld.20.0337.

99. Bhargava A, Shewade HD**.** The potential impact of the COVID-19 response related lockdown on TB incidence and mortality in India. Indian J Tuberc. 2020;67(4s):S139-46. doi: 10.1016/j.ijtb.2020.07.004.

100. Chatterjee S, Das P, Vassall A**.** Impact of COVID-19 restrictive measures on income and health service utilization of tuberculosis patients in India. BMC Infect Dis. 2022;22(1):711. doi: 10.1186/s12879-022-07681-z.

101. George S, Paranjpe A, Nagesh P, Saalim M**.** Barriers to treatment adherence for female tuberculosis (TB) patients during the COVID-19 pandemic: qualitative evidence from front-line TB interventions in Bengaluru City, India. Indian J Public Health. 2022;66(1):38-44. doi: 10.4103/ijph.ijph_1146_21.

102. Khan FMA, Kazmi Z, Hasan MM**.**  Resurgence of tuberculosis amid COVID‐19 in Peru: associated risk factors and recommendations. 2021;36(6):2441-2445. doi: 10.1002/hpm.3291.

103. Knipper M, Sedas AC, Keshavjee S, Abbara A, Almhawish N, Alashawi H, et al. The need for protecting and enhancing TB health policies and services for forcibly displaced and migrant populations during the ongoing COVID-19 pandemic. Int J Infect Dis. 2021;113 Suppl 1:S22-7. doi: 10.1016/j.ijid.2021.03.047.

104. Louie JK, Reid M, Stella J, Agraz-Lara R, Graves S, Chen L, et al. A decrease in tuberculosis evaluations and diagnoses during the COVID-19 pandemic. Int J Tuberc Lung Dis. 2020;24(8):860-2. doi: 10.5588/ijtld.20.0364.

105. McQuaid CF, Vassall A, Cohen T, Fiekert K, White RG**.** The impact of COVID-19 on TB: a review of the data. Int J Tuberc Lung Dis. 2021;25(6):436-46. doi: 10.5588/ijtld.21.0148.

106. Ryckman T, Robsky K, Cilloni L. Ending tuberculosis in a post-COVID-19 world: a person-centred, equity-oriented approach. Lancet Infect Dis. 2023;23(2):e59-66. doi: 10.1016/S1473-3099(22)00500-X..

107. Saunders MJ, Evans CA**.** COVID-19, tuberculosis and poverty: preventing a perfect storm. Eur Respir J. 2020;56(1):2001348. doi: 10.1183/13993003.01348-2020.

108. Shrinivasan R, Rane S, Pai M**.** India's syndemic of tuberculosis and COVID-19. BMJ Glob Health. 2020;5(11) doi: 10.1136/bmjgh-2020-003979.

109. Singh PK**.** Strengthening social protection for TB patients: Lessons from COVID-19. PLOS Global Public Health. 2022;2(8):e0000950. doi: 10.1371/journal.pgph.0000950.

110. Souza LLL, Santos FLd, Crispim JdA, Fiorati RC, Dias S, Bruce ATI, et al. Causes of multidrug-resistant tuberculosis from the perspectives of health providers: challenges and strategies for adherence to treatment during the COVID-19 pandemic in Brazil. BMC Health Serv Res. 2021;21(1):1-10. doi: 10.1186/s12913-021-07057-0.

111. Vanleeuw L, Zembe-Mkabile W, Atkins S**.** Falling through the cracks: Increased vulnerability and limited social assistance for TB patients and their households during COVID-19 in Cape Town, South Africa. PLOS Glob Public Health. 2022;2(7):e0000708. doi: 10.1371/journal.pgph.0000708.

112. Zumla A, Chakaya J, Khan M, Fatima R, Wejse C, Al-Abri S, et al. 'The Clock is Ticking'—and the world is running out of time to deliver the United Nations General Assembly commitments to End TB due to the COVID-19 pandemic. Int J Infect Dis. 2021;113(Suppl 1):S1-6. doi: 10.1016/j.ijid.2021.03.046.

113. Awan HA, Sahito AM, Sukaina M, Khatri G, Waheed S, Sohail F, et al. Tuberculosis amidst COVID-19 in Pakistan: a massive threat of overlapping crises for the fragile healthcare systems. Epidemiol Infect. 2022;150:e41. doi: 10.1017/s0950268822000358.

114. Cox V, Wilkinson L, Grimsrud A, Hughes J, Reuter A, Conradie F, et al. Critical changes to services for TB patients during the COVID-19 pandemic. Int J Tuberc Lung Dis. 2020;24(5):542-4. doi: 10.5588/ijtld.20.0205.

115. Driessche KV, Mahlobo PZ, Venter R**.** Face masks in the post-COVID-19 era: a silver lining for the damaged tuberculosis public health response? Lancet Respir Med. 2021;9(4):340-342. doi: 10.1016/S2213-2600(21)00020-5.

116. Jain VK, Iyengar KP, Samy DA, Vaishya R**.** Tuberculosis in the era of COVID-19 in India. Diabetes Metab Syndr. 2020;14(5):1439-43. doi: 10.1016/j.dsx.2020.07.034.

117. Jamal WZ, Habib S, Khowaja S, Safdar N, Zaidi SMA**.** COVID-19: ensuring continuity of TB services in the private sector. Int J Tuberc Lung Dis. 2020;24(8):870-2. doi: 10.5588/ijtld.20.0400.

118. Mannan S, Oga-Omenka C, Soman ThekkePurakkal A, Huria L, Kalra A, Gandhi R, et al. Adaptations to the first wave of the COVID-19 pandemic by private sector tuberculosis care providers in India. J Clin Tuberc Other Mycobact Dis. 2022;28:100327. doi: 10.1016/j.jctube.2022.100327.

119. Marti M, Zürcher K, Enane LA, Diero L, Marcy O, Tiendrebeogo T, et al. Impact of the COVID-19 pandemic on TB services at ART programmes in low- and middle-income countries: a multi-cohort survey. J Int AIDS Soc. 2022;25(10):e26018. doi: 10.1002/jia2.26018.

120. Masina HV, Lin IF, Chien LY**.** The Impact of the COVID-19 pandemic on tuberculosis case notification and treatment outcomes in Eswatini. Int J Public Health. 2022;67:1605225. doi: 10.3389/ijph.2022.1605225.

121. Andom AT, Fejfar D, Yuen CM, Ndayizigiye M, Mugunga JC, Mukherjee JS**.** The impact of COVID-19 on tuberculosis program performance in the Kingdom of Lesotho. Trop Med Infect Dis. 2023;8(3):165. doi: 10.3390/tropicalmed8030165.

122. Berra TZ, Ramos ACV, Alves YM, Tavares RBV, Tartaro AF, Nascimento MCD, et al. Impact of COVID-19 on tuberculosis indicators in Brazil: a time series and spatial analysis study. Trop Med Infect Dis. 2022;7(9):247. doi: 10.3390/tropicalmed7090247.

123. Bouaddi O, Hasan MM, Sahito AM, Shah PA, Mohammed AZA, Essar MY**.** Tuberculosis in the middle of COVID-19 in Morocco: efforts, challenges and recommendations. Trop Med Health. 2021;49(1):98. doi: 10.1186/s41182-021-00388-y.

124. Chiang CY, Islam T, Xu C, Chinnayah T, Garfin AMC, Rahevar K, et al. The impact of COVID-19 and the restoration of tuberculosis services in the Western Pacific Region. Eur Respir J. 2020;56(4):2003054. doi: 10.1183/13993003.03054-2020.

125. Cronin AM, Railey S, Fortune D, Wegener DH, Davis JB**.** Notes from the field: effects of the COVID-19 response on tuberculosis prevention and control efforts - United States, March-April 2020. MMWR Morb Mortal Wkly Rep. 2020;69(29):971-2. doi: 10.15585/mmwr.mm6929a4.

126. Dlangalala T, Musekiwa A, Brits A, Maluleke K, Jaya ZN, Kgarosi K, et al. . Evidence of TB services at primary healthcare level during COVID-19: a scoping review. Diagnostics (Basel). 2021;11(12):2221. doi: 10.3390/diagnostics11122221.

127. Fuady A, Houweling TAJ, Richardus JH**.** COVID-19 and Tuberculosis-Related Catastrophic Costs. Am J Trop Med Hyg. 2020;104(2):436-40. doi: 10.4269/ajtmh.20-1125.

128. Khan AW, Khan B, Shah SK, Kazi GN, Quadir A, Ghafoor A, et al. The impact of covid-19 on TB care in Pakistan during 2020. Pak J Public Health. 2022;12(1):8-11. doi: 10.32413/pjph.v12i1.997.

129. Khobragade RN, Kelkar RU, Sunilkumar M, Cency B, Murthy N, Surendran D, et al. Health system resilience: ensuring TB services during COVID-19 pandemic in Kerala, India. Indian J Tuberc. 2022;69(4):427-31. doi: 10.1016/j.ijtb.2021.10.004.

130. Kim B, Kang YA, Lee J. Heterogeneous impact of Covid-19 response on tuberculosis burden by age group. Sci Rep. 2022;12(1):13773. doi: 10.1038/s41598-022-18135-6.

131. Klinton JS, Oga-Omenka C, Heitkamp P**.** TB and COVID - Public and private health sectors adapt to a new reality. J Clin Tuberc Other Mycobact Dis. 2020;21:100199. doi: 10.1016/j.jctube.2020.100199.

132. Manhiça I, Augusto O, Sherr K, Cowan J, Cuco RM, Agostinho S, et al. COVID-19-related healthcare impacts: an uncontrolled, segmented time-series analysis of tuberculosis diagnosis services in Mozambique, 2017-2020. BMJ Glob Health. 2022;7(4):e007878. doi: 10.1136/bmjgh-2021-007878.

133. Mihika FA, Jubayer Biswas MAA, Khan MMH, Islam SS, Haque MA, Banu S, et al. The effect of the COVID-19 pandemic on pulmonary tuberculosis control in the selected Upazila health complexes of Dhaka Division, Bangladesh. Trop Med Infect Dis. 2022;7(11):385. doi: 10.3390/tropicalmed7110385.

134. Min J, Kim HW, Koo HK, Ko Y, Oh JY, Kim J, et al. Impact of COVID-19 pandemic on the National PPM Tuberculosis Control Project in Korea: the Korean PPM Monitoring Database between July 2019 and June 2020. J Korean Med Sci. 2020;35(43):e388. doi: 10.3346/jkms.2020.35.e388.

135. Nhari LG, Dzobo M, Chitungo I, Denhere K, Musuka G, Dzinamarira T**.** Implementing effective TB prevention and treatment programmes in the COVID-19 era in Zimbabwe. A call for innovative differentiated service delivery models. Public Health Pract (Oxf). 2020;1:100058. doi: 10.1016/j.puhip.2020.100058.

136. Oga-Omenka C, Sassi A, Vasquez NA, Baruwa E, Rosapep L, Daniels B, et al. Tuberculosis service disruptions and adaptations during the first year of the COVID-19 pandemic in the private health sector of two urban settings in Nigeria-A mixed methods study. PLOS Glob Public Health. 2023;3(3):e0001618. doi: 10.1371/journal.pgph.0001618.

137. Petersen E, Seif Al-Abr S, Chakaya J, Goletti D, Parolina L, Wejse C, et al. World TB Day 2022: Revamping and reshaping global TB control programs by advancing lessons learnt from the COVID-19 pandemic. Int J Infect Dis. 2022;124(Suppl 1):S1-3. doi: 10.1016/j.ijid.2022.02.057.

138. Reid MJA, Silva S, Arinaminpathy N, Goosby E. Building a tuberculosis-free world while responding to the COVID-19 pandemic. Lancet. 2020;396:1312-13. doi: 10.1016/S0140-6736(20)32138-3.

139. Roberts T, Sahu S, Malar J, Abdullaev T, Vandevelde W, Pillay YG, et al. Turning threats into opportunities: how to implement and advance quality TB services for people with HIV during the COVID-19 pandemic and beyond. J Int AIDS Soc. 2021;24(4):e25696. doi: 10.1002/jia2.25696.

140. Tran CH, Moore BK, Pathmanathan I, Lungu P, Shah NS, Oboho I, et al. Tuberculosis treatment within differentiated service delivery models in global HIV/TB programming. J Int AIDS Soc. 2021;24(Suppl 6):e25809. doi: 10.1002/jia2.25809..

141. van Rensburg AJ, Petersen I, Awotiwon A, Bachmann MO, Curran R, Murdoch J, et al. Applying learning health systems thinking in codeveloping integrated tuberculosis interventions in the contexts of COVID-19. BMJ Glob Health. 2022;7(10):e009567. doi: 10.1136/bmjgh-2022-009567.

142. Yadav P, Vohra C, Gopalakrishnan M, Garg MK**.** Integrating health planning and primary care infrastructure for COVID-19 and tuberculosis care in India: challenges and opportunities. Int J Health Plann Manage. 2022;37(2):632-42. doi: 10.1002/hpm.3393.

143. ACTION Global Health Advocacy Partnership et al. The impact of COVID-19 on the TB epidemic: a community perspective, 2020. Available from: <https://spark.adobe.com/page/xJ7pygvhrIAqW/>

144. Ferrer JP, Suzuki S, Alvarez C, Berido C, Caballero M, Caraig B, et al. Experiences, challenges and looking to the future in a clinical tuberculosis cohort in the time of COVID-19 in the Philippines. Trans R Soc Trop Med Hyg. 2021;115(6):579-82. doi: 10.1093/trstmh/trab025.

145. Loveday M, Cox H, Evans D, Furin J, Ndjeka N, Osman M, et al. Opportunities from a new disease for an old threat: Extending COVID-19 efforts to address tuberculosis in South Africa. S Afr Med J. 2020;110(12):1160-7. doi: 10.7196/SAMJ.2020.v110i12.15126.

146. Malik AA, Hussain H, Maniar R, Safdar N, Mohiuddin A, Riaz N, et al. Integrated tuberculosis and COVID-19 activities in Karachi and tuberculosis case notifications. Trop Med Infect Dis. 2022;7(1):12. doi: 10.3390/tropicalmed7010012.

147. Mandal A, Verma AK, Kar SK, Bajpai J, Kant S, Kumar S, et al. A cross-sectional study to determine the psychological distress among pulmonary tuberculosis patients during COVID-19 pandemic. Monaldi Arch Chest Dis. 2022;93(1) doi: 10.4081/monaldi.2022.2255.

148. Millones AK, Lecca L, Acosta D, Campos H, Del Águila-Rojas E, Farroñay S, et al. The impact of the COVID-19 pandemic on patients' experiences obtaining a tuberculosis diagnosis in Peru: a mixed-methods study. BMC Infect Dis. 2022;22(1):829. doi: 10.1186/s12879-022-07832-2.

149. Mwamba C, Kerkhoff AD, Kagujje M, Lungu P, Muyoyeta M, Sharma A**.** Diagnosed with TB in the era of COVID-19: patient perspectives in Zambia. Public Health Action. 2020;10(4):141-6. doi: 10.5588/pha.20.0053.

150. Santos FLD, Souza LLL, Bruce ATI, Crispim JA, Arroyo LH, Ramos ACV, et al. Patients' perceptions regarding multidrug-resistant tuberculosis and barriers to seeking care in a priority city in Brazil during COVID-19 pandemic: A qualitative study. PLoS One. 2021;16(4):e0249822. doi: 10.1371/journal.pone.0249822.

151. Sunjaya DK, Paskaria C, Pramayanti M, Herawati DMD, Parwati I. The magnitude of anxiety and depressive symptoms among tuberculosis patients in community health centers setting during the peak of COVID-19 pandemic. J Multidiscip Healthc. 2022;15:755-64. doi: 10.2147/jmdh.S359530.

152. Tinoco EM, Vasconcelos A, Alves F, Duarte R**.** Impact of COVID-19 on extrapulmonary TB and the benefit of decentralised TB services. Int J Tuberc Lung Dis. 2022;26(2):178-80. doi: 10.5588/ijtld.21.0675.

153. Togun T, Kampmann B, Stoker NG, Lipman M**.** Anticipating the impact of the COVID-19 pandemic on TB patients and TB control programmes. Ann Clin Microbiol Antimicrob. 2020;19(1):21. doi: 10.1186/s12941-020-00363-1.

154. Zhang G, Yu Y, Zhang W, Shang J, Chen S, Pang X, et al. Influence of COVID-19 for delaying the diagnosis and treatment of pulmonary tuberculosis-Tianjin, China. Front Public Health. 2022;10:937844. doi: 10.3389/fpubh.2022.937844.

155. Zimmer AJ, Heitkamp P, Malar J, Dantas C, O'Brien K, Pandita A, et al. Facility-based directly observed therapy (DOT) for tuberculosis during COVID-19: a community perspective. J Clin Tuberc Other Mycobact Dis. 2021;24:100248. doi: 10.1016/j.jctube.2021.100248.

156. Beyene NW, Sitotaw AL, Tegegn B, Bobosha K**.** The impact of COVID-19 on the tuberculosis control activities in Addis Ababa. Pan Afr Med J. 2021;38:243. doi: 10.11604/pamj.2021.38.243.27132.

157. Buonsenso D, Iodice F, Sorba Biala J, Goletti D**.** COVID-19 effects on tuberculosis care in Sierra Leone. Pulmonology. 2021;27(1):67-9. doi: 10.1016/j.pulmoe.2020.05.013.

158. Dheda K, Perumal T, Moultrie H, Perumal R, Esmail A, Scott AJ, et al. The intersecting pandemics of tuberculosis and COVID-19: population-level and patient-level impact, clinical presentation, and corrective interventions. Lancet Respir Med. 2022;10(6):603-22. doi: 10.1016/s2213-2600(22)00092-3.

159. Fatima R, Akhtar N, Yaqoob A, Harries AD, Khan MS**.** Building better tuberculosis control systems in a post-COVID world: learning from Pakistan during the COVID-19 pandemic. Int J Infect Dis. 2021;113(Suppl 1):S88-90. doi: 10.1016/j.ijid.2021.03.026..

160. Islam M**.** Extensively drug-resistant tuberculosis in the time of COVID-19-How has the landscape changed for Pakistan? Disaster Med Public Health Prep. 2020;14(4):e9-10. doi: 10.1017/dmp.2020.230.

161. Kumar P, Goyal JP**.** Tuberculosis during Covid-19 pandemic: challenges and opportunities. Indian Pediatr. 2020;57(11):1082. doi: 10.1007/s13312-020-2047-8.

162. Maroof M, Pamei G, Bhatt M, Awasthi S, Bahuguna SC, Singh P**.** Drug adherence to anti-tubercular treatment during COVID-19 lockdown in Haldwani block of Nainital district. Indian J Community Health. 2022;34(4):535-41. doi: 10.47203/IJCH.2022.v34i04.016.

163. Oga-Omenka C, Tseja-Akinrin A, Boffa J, Heitkamp P, Pai M, Zarowsky C**.** Commentary: Lessons from the COVID-19 global health response to inform TB case finding. Healthc (Amst). 2021;9(2):100487. doi: 10.1016/j.hjdsi.2020.100487.

164. Soko RN, Burke RM, Feasey HRA, Sibande W, Nliwasa M, Henrion MYR, et al. Effects of coronavirus disease pandemic on tuberculosis notifications, Malawi. Emerg Infect Dis. 2021;27(7):1831-9. doi: 10.3201/eid2707.210557.

165. Uwishema O, Badri R, Onyeaka H, Okereke M, Akhtar S, Mhanna M, et al. Fighting tuberculosis in Africa: the current situation amidst the COVID-19 pandemic. Disaster Med Public Health Prep. 2022;June 8:1-3. doi: 10.1017/dmp.2022.142.

166. Zimmer AJ, Klinton JS, Oga-Omenka C**.** Tuberculosis in times of COVID-19. J Epidemiol Community Health. 2022;76(3):310-6. doi: 10.1136/jech-2021-217529.

167. Anigbo AR, Gambhir L. Two years of the pandemic: Impact of COVID-19 on tuberculosis management in Nigeria. J Appl Pharm Sci. 2022;12(8):001-008. doi: 10.7324/japs.2022.120801.

168. Chapman HJ, Veras-Estévez BA**.** Lessons learned during the COVID-19 pandemic to strengthen tb infection control: a rapid review. Glob Health Sci Pract. 2021;9(4):964-77. doi: 10.9745/ghsp-d-21-00368.

169. Chen H, Zhang K**.** Insight into the impact of the COVID-19 epidemic on tuberculosis burden in China. Eur Respir J. 2020;56(3):2002710. doi: 10.1183/13993003.02710-2020.

170. Lipman M, McQuaid CF, Abubakar I, Khan M, Kranzer K, McHugh TD, et al. The impact of COVID-19 on global tuberculosis control. Indian J Med Res. 2021;153(4):404-8. doi: 10.4103/ijmr.IJMR_326_21.

171. Lungu PS, Kerkhoff AD, Muyoyeta M, Kasapo CC, Nyangu S, Kagujje M, et al. Interrupted time-series analysis of active case-finding for tuberculosis during the COVID-19 pandemic, Zambia. Bull World Health Organ. 2022;100(3):205-15. doi: 10.2471/blt.21.286109.

172. Nkereuwem O, Nkereuwem E, Fiogbe A, Usoroh EE, Sillah AK, Owolabi O, et al. Exploring the perspectives of members of international tuberculosis control and research networks on the impact of COVID-19 on tuberculosis services: a cross sectional survey. BMC Health Serv Res. 2021;21(1):798. doi: 10.1186/s12913-021-06852-z.

173. Rodrigues I, Aguiar A, Migliori GB, Duarte R**.** Impact of the COVID-19 pandemic on tuberculosis services. Pulmonology. 2022;28(3):210-9. doi: 10.1016/j.pulmoe.2022.01.015.

174. Shahnavazi M, Rigi F, Heydarikhayat N**.** Adherence to the treatment and influencing factors in patients with tuberculosis during the Covid-19 pandemic: a mixed method study. Health Educ Health Promot. 2022;10(4):633-42.

175. Tale S, Meitei Soibam P**.** Care of tuberculosis patients in the times of COVID-19. Indian J Tuberc. 2021;68(2):285-6. doi: 10.1016/j.ijtb.2020.09.004.

176. Wingfield T, Karmadwala F, MacPherson P, Millington KA, Walker NF, Cuevas LE, et al. Challenges and opportunities to end tuberculosis in the COVID-19 era. Lancet Respir Med. 2021;9(6):556-8. doi: 10.1016/s2213-2600(21)00161-2.
