# Supplementary material for: The global impact of COVID-19 on tuberculosis: A thematic scoping review, 2020–2023
Source: PLOS Glob Public Health. 2024 Jul 3;4(7):e0003043. doi: 10.1371/journal.pgph.0003043 (PMC11221697; doi:10.1371/journal.pgph.0003043)
Supplement: Attachment — Submitted filename: Response to Reviewers.docx [file pgph.0003043.s006.docx]

**Date:	19 May 2024**

**To:	*PLOS Global Public Heath* Editors & Peer Reviewers**

**From:	Michael H. Marco, Sevim Ahmedov, and Kenneth G. Castro**

**Re:	Comments on and Revisions to Manuscript #PGPH-D-24-00435**

*****

We appreciate the opportunity to reply to observations and comments by peer-reviewers and submit our revised manuscript, PGPH-D-24-00435,
“The Global Impact of COVID-19 on Tuberculosis: A Thematic Scoping Review, 2020-2023.”

A point-by-point table has been created, with the left column documenting the editors’ or peer reviewers’ requests and questions and the right column detailing the teams’ responses.

We’ve included the revised manuscript and supplemental material/grouped appendices with track changes and another with all changes/edits accepted.

We are at your disposal should additional revisions be required.

| **1. EDITORS’ SUGGESTIONS/COMMENTS** | | | |
| --- | --- | --- | --- |
|  | **QUESTION, COMMENT, OR CRITIQUE** |  | **RESPONSE** |
| 1. | “Please highlight the ethical considerations regarding usage of data in scoping review.” |  | Regarding the ethical considerations of using data in a scoping review, our team adheres to three essential factors:   - Researchers must ensure all personal information is kept confidential. - Sensitive or identifiable personal data should not be used in the publication or presentation of study data. - If the study involves human subjects, informed consent/ethical approval should be obtained.   **(Line 123)** |
| 2. | “Please assure the quality and clarity of the tables and pictures.” |  | The graphics have been recreated; they are clear and of high quality. |
| 3. | “The recommendations provided can help to develop TB care strategies in the emergence of another pandemic or a public health emergencies.” |  | We are in complete agreement. While it is unconventional to list policy recommendations in a scoping review, we felt that the subject warrants it. We mention their importance and utility several times. The last sentence in the “Background” sections states, “As COVID-19 continues to cause morbidity and mortality, and the threat of **future** airborne infection pandemics remains a real possibility, it seemed prudent to offer TB policy recommendations to global and national leaders to build and maintain a robust TB infrastructure with surge capacity for response, recovery, and resilience.” **(Line 113 as well as 553, 559, and 618)** To ensure the reader has quick access to the policy recommendations, they have been moved from the “Supplementary Materials” (grouped appendices) to the end of the “Results” section **(Line 448)** in the manuscript. |
| **2. Journal Requirements** | | | |
|  | **QUESTION/COMMENT/CRITIQUE** |  | **RESPONSE** |
| 1. | “Please review your reference list…” |  | Our references were carefully reviewed and updated during the initial editing process. There have been no changes since the initial submission of the manuscript. N.B. We complied with the instructions in the reference section of the “Submission Guidelines.” <https://journals.plos.org/globalpublichealth/s/submission-guidelines#loc-references> |
| 2. | Please send a completed 'Competing Interests' statement, including any COIs declared by your co-authors. If you have no competing interests to declare, please state "The authors have declared that no competing interests exist". Otherwise please declare all competing interests beginning with the statement "I have read the journal's policy and the authors of this manuscript have the following competing interests.” |  | No author has a “competing interest.” We have added the requested sentence, "The authors have declared that no competing interests exist."**(Line 39)** |
| 3. | “Please ensure that Funding Information and Financial Disclosure Statement are matched.” |  | This has been completed. Both read, “United States Agency for International Development provided salary support and publication fee.” **(Lines 33 & 36)** |
| 4. | “In “8” Funding Information you indicated that no funding was received. Please revise the Funding Information field to reflect funding received.” |  | This has been corrected. It now reads, “United States Agency for International Development provided salary support and publication fee.” **(Line 33)** |
| 5. | “We do not publish any copyright or trademark symbols that usually accompany proprietary names, eg  ©, ®, ™  (e.g. next to drug or reagent names). Please remove all instances of trademark/copyright symbols throughout the text, including  ® & ™ on pages 10 & 15.” |  | This has been corrected. **(Lines 143, 255, 256, and 365)** |

| **3.1. Reviewer 1’s “Comments to the Author”** | | |
| --- | --- | --- |
|  | **COMMENT/QUESTION** | **RESPONSE** |
| 1. | **Q:** “Does this manuscript meet PLOS Global Public Health’s [publication criteria](https://journals.plos.org/globalpublichealth/s/criteria-for-publication)? Is the manuscript technically sound, and do the data support the conclusions? The manuscript must describe methodologically and ethically rigorous research with conclusions that are appropriately drawn based on the data presented.”  A: “Yes” | Acknowledged, thank you |
| 2. | **Q: “**Has the statistical analysis been performed appropriately and rigorously?”  **A:** “N/A” | Acknowledged, thank you |
| 3. | **Q: “**Have the authors made all data underlying the findings in their manuscript fully available (please refer to the Data Availability Statement at the start of the manuscript PDF file)?” **A:** “Yes” | Acknowledged, thank you |
| 4. | **Q: “**Is the manuscript presented in an intelligible fashion and written in standard English?”  **A:** “Yes” | Acknowledged, thank you |
| **3.2. Reviewer 1’s “General Comments”** | | |
|  | **Comment/Question** | **Response** |
| 1. | “The manuscript presents a thematic scoping review to understand the global impact of COVID-19 on tuberculosis (TB) care and policy. It methodically categorizes the impacts into themes through a comprehensive review of selected studies, offering valuable insights into TB care during the pandemic and future preparedness. The topic is highly relevant and timely, considering the ongoing global health challenges posed by COVID-19 and TB. The authors have made a commendable effort to synthesize the available literature, and the manuscript aligns well with the PLOS Global Public Health scope.” | Acknowledged, thank you |
| **3.3. Reviewer 1’s “Specific Comment”** | | |
|  | **Comment/Question** | **Response** |
| 1. | 1. **Originality and Contribution to the Field:** “The manuscript successfully addresses a significant and timely public health issue. The thematic analysis approach provides a structured literature synthesis, contributing valuable insights. However, it would be beneficial to more explicitly highlight the novel contributions of this review in the introduction and conclusion sections, particularly about existing literature reviews on the topic.” | Thank you for your enthusiasm and belief that our manuscript significantly addresses an important global public health issue. We agree that we need to be more explicit about findings and ensuing policy recommendations in the review. To achieve this, 1) we have moved the recommendation out of the supplemental materials and into the manuscript with a discussion of their current and future importance; and 2) added a conclusion section to drive home the overarching significance of the thematic results. **(Line 612)** |
| 2. | **2. Methodology: “**The methodology section is well-structured, detailing the search strategy and selection criteria. However, the manuscript would benefit from a more detailed explanation of the thematic analysis process. Specifically, clarifying how themes were derived and refined during the review process could enhance the transparency and replicability of the study.” | Thank you for your comments on the “search strategy and selection criteria” of our thematic analysis. We discuss the thematic analysis process is the “Data Extraction and Quality Assessment” and “Data Synthesis and Analysis” sections as well as the presenting the “Data Extraction Instrument” (Appendix 1b; Table 1) that all three reviewers independently used to screen the publications to determine the themes in the review. Nevertheless, we agree that more clarification is needed and have added specific sentence on this topic in the “Methods” section. **(Line 160)** |
| 3. | **Statistical Analysis:** “This was not applicable as the exploratory scoping review did not need a meta-analysis.” | Acknowledged, thank you |
| 4. | **Results and Thematic Analysis: (1) “**The identification of ten principal themes is a major strength of this manuscript. Each theme is relevant and captures a critical aspect of the impact of COVID-19 on TB. Including illustrative quotes or examples from the literature is recommended to enrich the narrative and provide direct evidence for each theme.”  **(2**) “Consider presenting a summary table that maps the identified themes against the number of studies contributing to each theme, geographical distribution, and study design. This would give readers a quick overview of the evidence base supporting each theme**.”** | **(1)** Thank you for acknowledging that the ten themes are a major strength of the manuscript. We agree with your recommendation to enrich the narrative and have done so by synopsizing germane [themed] publications from a mix of counties and diverse populations that exemplify the impact on TB, be it causal factors or recommendations. At times we offer direct quotes, such as, “Dheda et al. [reference #180] (from the “Stigma” theme) shared their frank views of TB-related stigma in a 2022 commentary. They asserted that **“social stigma kills,”** noting that it is repugnant for people who battle airborne infectious diseases in isolation to fight social stigma simultaneously.” **(Line 415)** However, we predominantly paraphrase a specific authors’ remark(s) or assertion in their publication(s). This is exemplified in the “Case Notification and Finding” theme where it’s written, “Ruhwald, et al. [reference #53] **(Line 224)** emphasized that the novel use of COVID-19 molecular technologies and bi-directional testing will benefit TB diagnosis and reduce reliance on suboptimal tools, such as smear microscopy. Sahu, et al. [reference #7] asserted that these advanced diagnostics must be readily implemented in the community to meet people where they live.” **(Line 226)**  **2)** A summary table already exists in Supplementary Material [grouped appendices] **Appendix 3.** “Thematic Categorization of Publication [n=176].” Your helpful comment led us to reconstruct the table to demarcate the specific type of original research for over 100 publications in the review. |
| 5. | **(1) Discussion and Recommendations: “**The discussion provides a thoughtful interpretation of the findings within the broader TB care and policy context. The recommendations offered are pragmatic and relevant. To strengthen this section, the authors could consider discussing potential barriers and facilitators to implementing these recommendations in diverse health system contexts.”  **(2) “**It would be valuable to discuss the limitations of the scoping review methodology, such as the potential for publication bias and the exclusion of non-English language publications.” | Thank you for this useful suggestion. As mentioned above in point #3 in 1. EDITORS’ SUGGESTIONS/COMMENTS, we moved the policy recommendations out of the supplemental material and placed them in the end of the “Results” section of the manuscript. We also added a paragraph discussing the potential barriers and facilitators to implementing these recommendations. **(Line 555)**  (2) We agree: Researchers conducting a scoping review employing search engines to only search for publications in English create a bias as important non-English language data will neither be analyzed nor presented. This important limitation is acknowledged. |
| 6. | **Clarity, Language, and Structure:** “The manuscript is generally well-written and structured.” | Acknowledged, thank you. |
| 7. | **Ethics and Research Integrity:** “The manuscript adheres to high standards of research integrity. The authors have transparently reported their methodology and provided a comprehensive list of articles reviewed. A statement on the ethical considerations of using published literature in scoping reviews could further strengthen this aspect.” | Our team considers four issues regarding the ethical considerations of using published literature in a scoping review: 1) Accurate citation and attribution; 2) Bias and conflicts of interest; 3) Source quality and dependability; and 4) Defining scope and ensuring relevance.  In addition, most (if not all) of cited journals adhere to the International Committee of Medical Journal Editors (ICMJE) recommendation that require peer reviewed publications to disclose issues of informed consent, IRB and/or ethical committee approval in their methods section. |
| 8. | **Reporting Guidelines and Data Availability:** “The authors conducted the review in line with PRISMA-Scr.”  (1) “A PRISMA-ScR checklist could be included as supplementary material.”  (2) “A detailed supplementary section was included with the submission.” | (1) Thank you for your suggestions. Please note a PRISMA-ScR checklist is in the original submission. It is in “Appendix 2” of the “Supplementary Material.”  (2) Acknowledged, thank you |
|  | **Conclusion:** “This scoping review makes a valuable contribution to understanding the multifaceted impact of COVID-19 on TB care and policy. With minor revisions and enhancements, particularly in clarifying the thematic analysis process and enriching the discussion of findings, this manuscript will be a significant addition to the literature. The recommendations provided can inform future TB care strategies in the context of global health emergencies.” | Thank you for thoughtful suggestions on enhancing and emphasizing the importance and timeliness of the research topic, our thematic results, and ensuing policy recommendations. Your suggestions have convinced us to add a conclusion section to emphasize and enrich our findings and recommendations. **(Line 612)** |

| **3.2. Reviewer 2’s “Comments to the Author”** | | |
| --- | --- | --- |
|  | **COMMENT** | **RESPONSE** |
| 1. | **Q:** “Does this manuscript meet PLOS Global Public Health’s [publication criteria](https://journals.plos.org/globalpublichealth/s/criteria-for-publication)? Is the manuscript technically sound, and do the data support the conclusions? The manuscript must describe methodologically and ethically rigorous research with conclusions that are appropriately drawn based on the data presented.”  A: “Yes” | Acknowledged, thank you |
| 2. | **Q: “**Has the statistical analysis been performed appropriately and rigorously?”  **A:** “I don’t know.” | Thank you for your comment. According to Tricco, et al. from PRISMA (reference #11 in the manuscript), statistical analysis is not necessary because a scoping review is “type of knowledge synthesis that follows a systematic approach to map evidence on a topic and identify main concepts, theories, sources, and knowledge gaps.” We believe the answer would be N/A. |
| 3. | **Q: “**Have the authors made all data underlying the findings in their manuscript fully available (please refer to the Data Availability Statement at the start of the manuscript PDF file)?”  **A:** “Yes” | Acknowledged, thank you |
| 4. | **Q: “**Is the manuscript presented in an intelligible fashion and written in standard English?”  **A:** “Yes” | Acknowledged, thank you |
| **3.3. Reviewer 2’s “Specific Comments”** | | |
|  | **COMMENT** | **RESPONSE** |
| 1. | “At data sources and researches ; authors use few Mesh term such as not remembered another terms of covid-19 such as 2019 nCOV disease , coronavirus disease 2019, 2019 nCOV infection , Coronavirus disease 2019,2019 novel coronavirus disease as the term covid-19 is considered late by WHO as at onset of pandemic there is many terms not standardized with global term and also i noticed also you remembered at section of methods your objective of the study is The analysis of publications and grey literature was conducted to identify and define resonating  themes of COVID-19’s global impact on TB and inform policy recommendations to assist in TB  resilience, recovery, and future pandemic preparedness.” | Thank you for this comment. We were aware that the fundamentally essential, least amount of mesh terms would yield us a greater number of publications from the three search engines. We strove for this. Our goal was to cast the widest net **(Line 582),** to capture even the most remotely relevant publication to analyze so that as many voices from as many counties would be represented. Our initial cache from the search engines was 1755 publications **(Line 179)** In addition, none of our publications were from 2019. Our earliest publications were published in the first few months of 2020 when COVID and/or COVID-19 were becoming predominant terms for the virus. |
| 2. | You used limited MESH terms and not remember at your research terms regarding extra pulmonary tuberculosis or preventive measure like missed cases of BCG vaccines as at lock-down there are missed births or neonates not vaccinated or late at intake of BCG vaccine and also missed notification at diagnosis. | Thank you for your comment. Yes, we were interested in learning the impact of COVID-19 on BCG vaccination, extra pulmonary TB, etc. We, however, narrowed our scope (e.g., mesh terms) for two reasons: 1) by simply listing ‘mycobacterium tuberculosis,’ ‘tuberculosis,’ and ‘TB,’ we were certain that these topics would pick up all of those issues that are part of TB’s cascade of care; and 2) the themes in our thematic analysis needed to originate from what came from the search engines in using the above terms. To put in terms, such a TB diagnostics, TB prevention, etc., would have biased our search and the sample of publications. |
| 3. | “Pictures at manuscript are unclear and unfortunately not able to check or review it.” | Yes, you are correct, and we apologize that you were unable to clearly view our graphics. However, we recreated the graphics and to ensure their clarity and readability. |
| 4. | PLOS authors have the option to publish the peer review history of their article ([what does this mean?](https://journals.plos.org/globalpublichealth/s/editorial-and-peer-review-process#loc-peer-review-history)). If published, this will include your full peer review and any attached files | All authors of our scoping review agree to have *PLOS Global Public Health* publish the peer review history. |
